# Good and Bad Filters

**Published:** 1912-12

**Authors:** Wm. G. Bissell


### Good and Bad Filters

A great many inquiries have been made of the Bureau of Bacteriology regarding the efficacy of water filters. Unfortunately, the majority of such devices, as operated in individual households, do not render the water absolutely safe for use. There are filters which, when properly installed and operated, will remove bacteria. The mere attaching of a filter to a water supply and the ocassional scrubbing of the inner cylinder does not insure the rendition of a pure water. A great many such devices are, at the present time, in general use, the individual user having a false feeling of safety.

The laboratory will test the morking efficiency of any individual water filter upon the request of the resident of the city of Buffalo having the filter.

As this may be a time taking procedure, the requests should be made in writing, direct to the Bureau of Bacteriology, which division will perform the work at the earliest possible moment.

DR. WM. G. BISSELL.

